# Epidemiological characterization of rare diseases in Brazil: A retrospective study of the Brazilian Rare Diseases Network

**DOI:** 10.1186/s13023-024-03392-7

**Published:** 2024-10-30

**Authors:** Bibiana Mello de Oliveira, Filipe Andrade Bernardi, João Francisco Baiochi, Mariane Barros Neiva, Milena Artifon, Alberto Andrade Vergara, Ana Maria Martins, Anete Sevciovic Grumach, Angelina Xavier Acosta, Antonette Souto El Husny, Bethania de Freitas Rodrigues Ribeiro, Camila Ferreira Ramos, Carlos Eduardo Steiner, Chong Ae Kim, Denise Maria Christofolini, Diego Bettiol Yamada, Ellaine Doris Fernandes Carvalho, Erlane Marques Ribeiro, Fabíola de Arruda Bastos, Faradiba Sarquis Serpa, Flávia Reseda Brandão, Giselle Maria Araujo Felix Adjuto, Isabelle Carvalho, Jonas Alex Morales Saute, Juan Clinton Llerena Junior, Larissa Souza Mario Bueno, Luiz Carlos Santana da Silva, Mara Lucia Schmitz Ferreira Santos, Marcela Câmara Machado Costa, Marcia Maria Costa Giacon Giusti, Marcial Francis Galera, Márcio Eloi Colombo Filho, Maria Denise Fernandes Carvalho de Andrade, Maria Teresinha De Oliveira Cardoso, Marilaine Matos de Menezes Ferreira, Michelle Zeny, Milena Coelho Fernandes Caldato, Ney Boa Sorte, Nina Rosa de Castro Musolino, Paula Frassinetti Vasconcelos de Medeiros, Paulo Ricardo Gazzola Zen, Raquel Tavares Boy Da Silva, Rayana Elias Maia, Rodrigo Fock, Rosemarie Elizabeth Schimidt Almeida, Solange Oliveira Rodrigues Valle, Tatiana Amorim, Thaís Bomfim Teixeira, Vania Mesquita Gadelha Prazeres, Victor Evangelista de Faria Ferraz, Vinicius Costa Lima, Wagner José Martins Paiva, Ida Vanessa Doederlein Schwartz, Domingos Alves, Têmis Maria Félix, Raras Network Group, Adlya de Sousa Melo, Adrya Rafaela da Silva Rocha, Amanda Aragão, Amanda Delfino Braccini, Amanda Maria Schmidt, Ana Mondadori dos Santos, Ana Carolina de Souza e Silva, Ana Catarina Góes Leite Lima, Anna Luiza Scasso, Anne Caroline Magalhães Oliveira, Arthur Perico, Bárbara da Silva Aniceto, Barbara Pinheiro, Beatriz Ono Badaró, Beatriz Brasil Braga, Beatriz de Oliveira Chapiesk, Beatriz Felix Pinheiro, Beatriz Pereira, Betânia de Souza Ponce, Bianca Martins, Blenda Antunes Cacique Curçino de Eça, Bruna de Souza, Brunno Busnardo Paschoalino, Bruno Valadares, Caio Lôbo de Oliveira, Camila Sales, Carine Pacheco Alexandre, Carla Desengrini Girelli, Carolina Balluz, Carolina de Paiva Farias, Carolina Oliveira Vilemar, Caroline Duarte Arrigoni, Catharina de Almeida Passos, Catharine Harumi, Cleber Barbieri, Daniel Prado, Daniela Monteiro, Dhallya Andressa da Silva Cruz, Eduardo Batista, Eduardo José Pereira Naves, Elaine Samara Pinheiro Mendes da Silva, Estela Teixeira, Fabio Amaral, Fernanda Caroline Moreira, Flavia Liberato de Souza, Flavia Boggian, Francisco André Gomes Bastos Filho, Gabriel Lima Lôla, Gabriel Pereira, Gabrielle Diehl, Giovanna Pessanha Cordeiro, Giulia Duran, Gustavo Foz Fonseca, Helena Mello, Henrique Serpa, Henrique Veiga, Ingrid Gabriel, Isabella Formenti, Isabella de Brito Ramos, Isabella Ramos Paiva, Janaina Ferreira, Jannine Barboza Rangel, Jôbert Pôrto Florêncio, Josevaldo Monteiro Maia Filho, Júlia Emily Silva Dantas, Julia Cordeiro Milke, Juliana Rios, Julya Pavao, Kahue Aluaxe Angelo, Karina Montemor Klegen de Oliveira, Katheryne Barbosa de Carvalho, Kauanne Zulszeski, Leticia Raabe Mota de Lima, Livia Polisseni Cotta Nascimento, Lorena Alves dos Santos Pereira, Lorenzo Makariewicz, Luan Junio Pereira Bittencourt, Luana Medeiros, Luana Souza Vasconcelos, Lucca Nogueira Paes Jannuzzi, Luciana Costa Pinto da Silva, Luisa Aguilar, Luiza Valeria Chibicheski, Luiza de Oliveira Simões, Maria Teresa Aires Cabral Dias, Mariana Lopes dos Santos, Mariana Pacheco Oliveira Neves, Marina Teixeira Henriques, Matheus Viganô Leal, Milena Atique Tacla, Milena Soares Souza, Moises Ribeiro da Paz, Morya Silva, Natan Soares, Nicole da Silva Gilbert, Otavio Mauricio Silva, Paula Dourado Sousa, Paulo Rocha, Raissa Emanuelle Jacob, Raissa Vieira Leite da Silva, Raniery Barros Carvalho, Raphaella Nagib Carvalho Santos, Raquel Silva, Rebeca Pedrosa Holanda, Rebeca Falcão Lopes Mourão, Ricardo Cunha de Oliveira, Rodrigo Mesquita Costa Braga, Sabrina Macely, Sergio Morais, Sheila Constância Adolfo Mabote Mucumbi, Simei Nhime, Stefanny Karla Ferreira de Sousa, Tauane Franca Rego, Thayane Holanda Gurjão, Thuanne Cidreira dos Santos Gomes, Tiago Ramos Gazineu, Victória Scheibe Machado, Victória Feitosa Muniz, Victória Rocha, Vitor Leão, Wendyson Oliveira, Willian Miguel, Yasmin de Araújo Ribeiro, Yasmin Amorim dos Santos

**Affiliations:** 1 Medical Genetics Service, Hospital de Clínicas de Porto Alegre, Porto Alegre, Brazil; 2 Postgraduation Program in Genetics and Molecular Biology, Federal University of Rio Grande Do Sul, Porto Alegre, RS Brazil; 3 Engineering School of São Carlos, Bioengineering Department, University of São Paulo, São Carlos, SP Brazil; 4 Ribeirão Preto Medical School, University of São Paulo, Ribeirão Prêto, SP Brazil; 5 Institute of Mathematics and Computer Sciences, São Carlos Campus, University of São Paulo, São Carlos, SP Brazil; 6 Medical Genetics Service, Hospital de Clínicas de Porto Alegre, Porto Alegre, RS Brazil; 7 Hospital Infantil João Paulo II, Belo Horizonte, MG Brazil; 8 Hospital São Paulo, São Paulo, SP Brazil; 9 Faculdade de Medicina do Centro Universitario FMABC, Santo André, SP Brazil; 10 Hospital Universitário Prof. Edgar Santos and Faculdade de Medicina da Bahia da Universidade Federal da Bahia, Salvador, BA Brazil; 11 Hospital Universitário Bettina Ferro de Souza, Universidade Federal Do Pará, Belém, PA Brazil; 12 Fundação Hospital Estadual do Acre, Rio Branco, AC Brazil; 13 Hospital Universitário Prof. Edgar Santos, Salvador, BA Brazil; 14 Universidade Estadual de Campinas, Campinas, SP Brazil; 15 Instituto da Criança, Faculdade de Medicina da Universidade de São Paulo, São Paulo, SP Brazil; 16 Ribeirao Preto Medical School, University of Sao Paulo, Ribeirão Prêto, SP Brazil; 17 Hospital Geral Dr. César Cals, Fortaleza, CE Brazil; 18 Hospital Infantil Albert Sabin, Fortaleza, CE Brazil; 19 Centro Universitário do Estado do Pará, Belém, PA Brazil; 20 Hospital Santa Casa de Misericórdia de Vitória, Vitória, ES Brazil; 21 Centro de Diabetes e Endocrinologia da Bahia, Salvador, BA Brazil; 22 Hospital de Apoio de Brasília, Brasília, DF Brazil; 23 Universidade Federal do Rio Grande do Sul, Porto Alegre, RS Brazil; 24 Instituto Nacional de Saúde da Mulher, da Criança e do Adolescente Fernandes Figueira/Fiocruz, Rio de Janeiro, RJ Brazil; 25 Maternidade Climério de Oliveira, Salvador, BA Brazil; 26 Hospital Pequeno Príncipe, Curitiba, PR Brazil; 27 Escola Bahiana de Medicina e Saúde Pública, Salvador, BA Brazil; 28 Instituto Jô Clemente, São Paulo, SP Brazil; 29 Hospital Universitário Júlio Müller, Cuiabá, MT Brazil; 30 Hospital Universitário Walter Cantídio, Universidade Estadual do Ceará, Fortaleza, CE Brazil; 31 Hospital Materno Infantil de Brasília, Brasília, DF Brazil; 32 Centro Universitário do Pará, Belém, PA Brazil; 33 Instituto de Psiquiatria Hospital das Clínicas da Faculdade de Medicina da Universidade de São Paulo, São Paulo, SP Brazil; 34 Unidade Acadêmica de Medicina, Centro de Ciências Biológicas e de Saúde, Hospital Universitário Alcides Carneiro, Universidade Federal de Campina Grande, Campina Grande, PB Brazil; 35 Hospital da Criança Santo Antônio, Universidade Federal de Ciências da Saúde de Porto Alegre, Porto Alegre, RS Brazil; 36 Hospital Universitário Pedro Ernesto, Rio de Janeiro, RJ Brazil; 37 Hospital Universitário Lauro Wanderley, João Pessoa, PB Brazil; 38 Universidade Estadual de Londrina, Londrina, PR Brazil; 39 Hospital Universitário Clementino Fraga Filho, Rio de Janeiro, RJ Brazil; 40 Associação de Pais e Amigos dos Excepcionais de Salvador, Salvador, BA Brazil; 41 Associação de Pais e Amigos dos Excepcionais de Anápolis, Anápolis, GO Brazil; 42 Policlínica Codajás, Manaus, AM Brazil; 43 Hospital das Clínicas da Faculdade de Medicina de Ribeirão Preto da Universidade de São Paulo, Ribeirão Prêto, SP Brazil; 44 Health Intelligence Laboratory, Ribeirão Preto Medical School, University of São Paulo, Ribeirão Prêto, SP Brazil; 45 Department of Social Medicine, Ribeirão Preto Medical School, University of São Paulo, Ribeirão Prêto, SP Brazil; 46 Medical Genetics Service, Hospital de Clínicas de Porto Alegre, Rua Ramiro Barcelos, 2350, Porto Alegre, RS 90035-903 Brazil

**Keywords:** Rare diseases, Public Health System, Brazil, Brazilian Rare Diseases Network

## Abstract

**Background:**

The Brazilian Policy for Comprehensive Care for People with Rare Diseases was implemented in 2014; however, national epidemiological data on rare diseases (RDs) are scarce and mainly focused on specific disorders. To address this gap, University Hospitals, Reference Services for Neonatal Screening, and Reference Services for Rare Diseases, all of which are public health institutions, established the Brazilian Rare Diseases Network (RARAS) in 2020. The objective of this study was to perform a comprehensive nationwide epidemiological investigation of individuals with RDs in Brazil. This retrospective survey collected data from patients receiving care in 34 healthcare facilities affiliated with RARAS in 2018 and 2019.

**Results:**

The survey included 12,530 participants with a median age of 15.0 years, with women representing 50.5% of the cohort. Classification according to skin color demonstrated that 5044 (47.4%) participants were admixed. Most had a confirmed diagnosis (63.2%), with a predominance of phenylketonuria (PKU), cystic fibrosis (CF), and acromegaly. Common clinical manifestations included global developmental delay and seizures. The average duration of the diagnostic odyssey was 5.4 years (± 7.9 years). Among the confirmed diagnoses, 52.2% were etiological (biochemical: 42.5%; molecular: 30.9%), while 47.8% were clinical. Prenatal diagnoses accounted for 1.2%. Familial recurrence and consanguinity rates were 21.6% and 6.4%, respectively. Mainstay treatments included drug therapy (55.0%) and rehabilitation (15.6%). The Public Health System funded most diagnoses (84.2%) and treatments (86.7%). Hospitalizations were reported in 44.5% of cases, and the mortality rate was 1.5%, primarily due to motor neuron disease and CF.

**Conclusion:**

This study marks a pioneering national-level data collection effort for rare diseases in Brazil, offering novel insights to advance the understanding, management, and resource allocation for RDs. It unveils an average diagnostic odyssey of 5.4 years and a higher prevalence of PKU and CF, possibly associated with the specialized services network, which included newborn screening services.

**Supplementary Information:**

The online version contains supplementary material available at 10.1186/s13023-024-03392-7.

## Introduction

Rare diseases (RDs) are individually rare but collectively affect a significant proportion of the population. Approximately 71.9% of RDs have a genetic cause, and there are over 6000 known RDs [1]. They represent a serious public health problem with major unmet needs since many are life-limiting or chronically debilitating. Patients and families with RDs often face long diagnostic journeys, while healthcare professionals struggle with identifying, managing, and obtaining accurate information about these conditions. RDs are often associated with early mortality and a considerable reduction in quality of life [1–5].

In Brazil, the Ministry of Health defines an RD as any disorder that affects up to 65 per 100,000 individuals [3, 4]. Previous international studies have reported an estimated population prevalence of RDs of 3.5–8.0%, suggesting that they have a substantial impact on public health [1, 5, 6]. Extrapolating these estimates to the Brazilian population [7] produces a corresponding figure of 7.0–16.2 million Brazilians affected by RDs, highlighting their significant burden and public health implications.

Brazil, the fifth-largest country worldwide, covers 8,510,417 square kilometers and is divided into five regions with 26 states, a Federal District, and 5570 municipalities [7]. The Brazilian Unified Health System (*Sistema Único de Saúde* [SUS]) was established in 1988 and aims to provide universal and equitable access to promotion, prevention, and health care services for all Brazilian citizens. Brazil has undergone an epidemiological transition in recent decades, marked by significant advancements in health indicators attributable to external factors. Notably, hereditary diseases and congenital anomalies contribute significantly to child mortality, ranking second among infant mortality causes since 2005 [8, 9].

In January 2014, the Brazilian Policy for Comprehensive Care for Persons with Rare Diseases was established within the scope of the SUS. This policy aims to reduce morbidity and mortality and improve the quality of life of individuals with RDs through promotion, prevention, early detection, timely treatment, disability reduction, and palliative care. It classifies RDs as genetic and non-genetic, with genetic RDs grouped into three categories: congenital anomalies and late-onset disorders, intellectual disability, and inborn errors of metabolism [10].

To date, over 30 reference services for RDs have been accredited. This is still insufficient to meet population demands. Most cases are treated in university hospitals (UHs), but whether their human and technological resources are adequate for RD care is unknown [10, 11]. Despite advances in diagnosis, mainly due to the development of new technologies and the recent organization of RD care in Brazil, the country lacks an established system for registering RDs. Except for a few infectious RDs that require mandatory reporting, epidemiological data on these conditions are scarce and, when available, are often restricted to specific RDs [2, 3].

High-quality epidemiological data on RDs are essential for understanding patient needs, enhancing healthcare management, and identifying the potential beneficiaries of clinical trials and novel therapies. However, epidemiological research encounters obstacles since many studies rely on limited national registries that often focus on specific disease groups [5]. Therefore, a coordinated effort to map the epidemiology of RDs in Brazil is needed. The Brazilian Rare Diseases Network (RARAS) was established in 2020 to bridge this gap, including UHs, RD reference services (RDRSs), and newborn screening reference services (NSRSs). This initiative encompasses a national survey of the epidemiology, diagnosis, clinical presentation, and treatment of individuals with genetic and non-genetic RDs. It has two phases: retrospective and prospective. The retrospective phase involved data collection on RD cases treated at centers in 2018 and 2019, while data collection for the prospective phase has been going on since 2022 [2, 3]. This study presents the findings of the retrospective phase, undertaking a comparative analysis of distinct diagnostic status groups.

## Materials and methods

A retrospective survey was conducted to collect data from patients under diagnostic investigation or with a diagnosis or suspicion of an RD who were evaluated between 2018 and 2019 at 34 centers participating in the RARAS. These centers include 15 UHs, 4 RDRSs, and 3 NSRSs, with the remaining centers having mixed roles: 8 are both an RDRS and a UH, 3 are both an RDRS and an NSRS, and 1 is both an NSRS and a UH. A map of the participating centers can be seen in Additional File 1.

This project’s methodology has been previously published by Alves et al. [2] and Félix et al. [3]. All participating network services retrospectively searched for cases with genetic and non-genetic RDs and those under diagnostic investigation. Researchers collected data from each service by accessing medical records, using a standardized form in the Research Electronic Data Capture (REDCap) platform hosted at Ribeirao Preto Medical School, University of São Paulo [12]. The original survey is available at LattesData [13]. The form collected demographic, clinical, and therapeutic data. Given the different backgrounds of the data collectors, training was conducted for the participating centers. Initially, a pilot project was performed in five centers with different medical record management forms (paper or electronic). Two hundred and fifty cases were collected during the pilot phase from December 7, 2020, to January 15, 2021. The data were validated and curated. Based on this validation, retrospective data collection was initiated in the centers, which ended in March 2022.

Skin color was described according to the Brazilian Institute of Geography and Statistics (IBGE) as *parda* (admixed), *branca* (white), *preta* (black), *amarela* (yellow), and *indígena* (indigenous). Phenotypic data were described according to the Human Phenotype Ontology (HPO) [14] and limited to five terms per case. Diagnostic information was recorded based on international ontologies (International Statistical Classification of Diseases and Related Health Problems, Tenth Revision [ICD-10] [15]; Orphanet [ORPHA] [16]; or Online Mendelian Inheritance in Man [OMIM] [17]), enabling comparison and aggregation with Orphadata. Reasons for hospitalization and causes of death were documented using ICD-10 [3].

Data analyses were performed using the IBM® SPSS Statistics software (version 26) and Python language (version 3.9.17), leveraging the Pandas (version 1.5.3), NumPy (version 1.24.3), and SciPy (version 1.10.1) libraries. In the descriptive analyses, each individual was evaluated independently. In the comparative analyses based on diagnostic status, each diagnosis was considered independently, as an individual might have more than one RD diagnosis. The chi-squared test was used to compare nominal variables, while the Kruskal–Wallis test was applied to compare continuous numerical variables. In both cases, the Bonferroni correction was utilized for multiple comparisons. The significance level was set at 0.05.

## Results

### Population

**Fig. 1:**
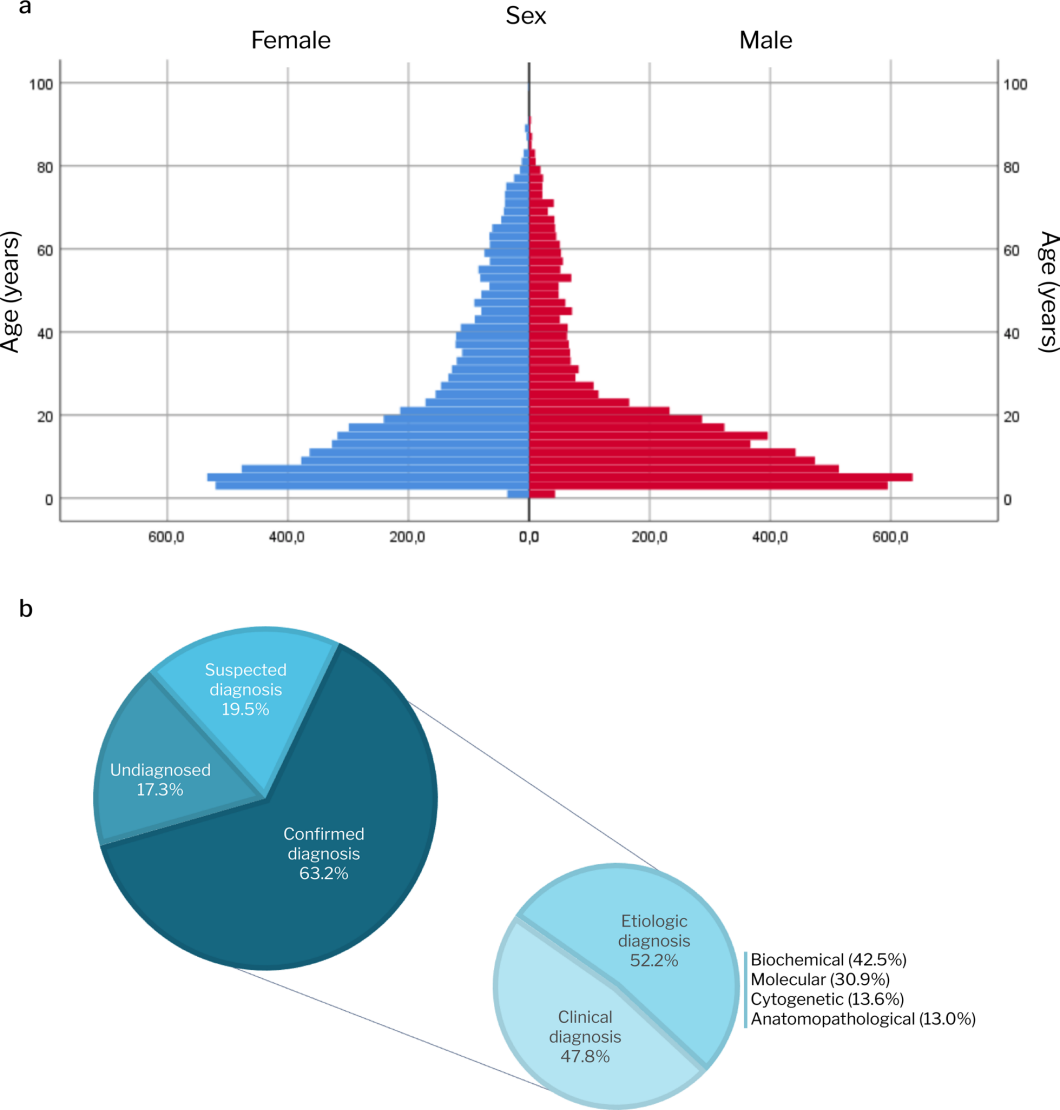
**a** Histogram of participants’ age and sex distribution (*n* = 12,502) and **b** diagnostic status (*n* = 12,279)

Data from 12,530 participants across 34 centers were collected. Most of the sample was female (*n* = 6331; 50.6%), and 13 (0.1%) individuals had undetermined sex. The median age was 15.0 years (interquartile range [IQR]: 7–31; mean: 24.9 ± 20.4; range: 1–98) at the time of inclusion (Fig. 1a). The sample’s characteristics are shown in Table 1.

**Table 1:** Sample characteristics (*n* = 12,530)

	N	%
*Color or race*		
Admixed	5044	47.5
White	4881	45.9
Black	609	5.7
Yellow	68	0.6
Indigenous	30	0.3
*Sex*		
Female	6331	50.6
Male	6171	49.3
Undetermined	13	0.1
*Region of birth*		
Southeast	3765	33.6
Northeast	3729	33.2
South	1659	14.8
Midwest	1377	12.3
North	673	6.0
Born in other countries	12	0.1
*Region of residence*		
Southeast	3996	32.8
Northeast	3950	32.5
South	2081	17.1
Midwest	1497	12.3
North	642	5.3

Classification according to skin color demonstrated that 5044 (47.5%) individuals were admixed and 4881 (45.9%) were white. Most participants were born in the Southeast (*n* = 3765; 33.6%) and Northeast (*n* = 3729; 33.2%) regions. Individuals born in 1750 Brazilian municipalities were included. Twelve participants (0.1%) were born in other countries: two in Lebanon and one each in Egypt, Ecuador, Guinea-Bissau, Japan, Paraguay, Peru, Portugal, and Venezuela (Table 1). Most participants lived in the Southeast region (*n* = 3996; 32.8%), followed by the Northeast region (*n* = 3950; 32.5%).

The first evaluation at the participating centers occurred at a median age of 6.2 years (IQR: 0.9–20.7). The participants had a median follow-up duration of 2.8 years in the centers (IQR: 0.6–7.9) and 1.7 years in the medical specialty (IQR: 0.1–1.7). Of the total sample, 92 participants were followed up in more than one participating center.

### Diagnosis

Regarding diagnosis status, 7931 (63.2%) participants had a confirmed diagnosis, while 2450 (19.5%) had a suspected diagnosis, and 2177 (17.3%) were considered undiagnosed. Sixty-seven participants had more than one confirmed RD diagnosis: 65 had two, and two had three.

Regarding the diagnostic terminology, 6644 (64.7%) of the diagnoses were recorded using an ORPHA code, 2794 (27.2%) using an ICD-10 code, and 825 (8.0%) using an OMIM code. A total of 1778 different diagnostic codes were mentioned. The most frequent diseases were phenylketonuria (PKU; *n* = 623), cystic fibrosis (CF; *n* = 506), and acromegaly (*n* = 382; Table 2). The diagnostic codes aggregated for the ten most prevalent conditions are detailed in Additional File 2. Upon excluding cases diagnosed through newborn screening, the most frequent diagnoses were CF (*n* = 389), acromegaly (*n* = 381), and osteogenesis imperfecta (*n* = 361). The distribution of the most frequently reported diagnostic codes at each participating center is detailed in Additional file 3.

**Table 2:** The ten most frequent disorders, signs and symptoms, causes of hospitalization, and causes of death

Most frequent diagnoses (N =12,261)*			
	Description	N	%
	Phenylketonuria	623	5.1
	Cystic Fibrosis	506	4.1
	Acromegaly	382	3.1
	Osteogenesis Imperfecta	360	2.9
	Dystrophinopathy	278	2.3
	Congenital adrenal hyperplasia	275	2.2
	Neurofibromatosis	271	2.2
	Mucopolysaccharidosis	225	1.8
	Amyotrophic lateral sclerosis	211	1.7
	Turner Syndrome	197	1.6

Most frequent signs and symptoms (N = 34,685)**			
HPO	Description	N	%
HP:0001263	Global developmental delay	1246	3.6
HP:0001250	Seizure	734	2.1
HP:0004322	Short stature	678	2.0
HP:0001249	Intellectual disability	514	1.5
HP:0001252	Hypotonia	451	1.3
HP:0005982	Reduced phenylalanine hydroxylase level	391	1.1
HP:0001324	Muscle weakness	390	1.1
HP:0002315	Headache	331	0.9
HP:0000252	Microcephaly	326	0.9
HP:0002015	Dysphagia	298	0.8

Most frequent causes of hospitalization (N = 4,922)***			
ICD-10	Description	N	%
E22.0	Acromegaly and pituitary gigantism	189	3.8
Q78.0	Osteogenesis imperfecta	161	3.3
E84	Cystic fibrosis	125	2.5
J18–J18.9	Pneumonia, organism unspecified	119	2.4
G12.2	Motor neuron disease	87	1.8
E25	Adrenogenital disorders	50	1.0
E84.0	Cystic fibrosis with pulmonary manifestations	46	0.9
R56	Convulsions, not elsewhere classified	38	0.8
G71.0	Muscular dystrophy	33	0.7
G40	Epilepsy and recurrent seizures	32	0.6

Most frequent causes of death (N = 177)			
ICD-10	Description	N	%
G12.2	Motor neuron disease	28	15.8
E84	Cystic fibrosis	10	5.6
I46	Cardiac arrest	7	3.9
R09.2	Respiratory arrest	3	1.7
J96.9	Respiratory failure, unspecified	3	1.7
J96.0	Acute respiratory failure	3	1.7
J96	Respiratory failure, not elsewhere classified	3	1.7
J38.4	Edema of larynx	2	1.1
E74.0	Glycogen storage disease	2	1.1
A41.9	Sepsis, unspecified organism	2	1.1
A41	Other sepsis	2	1.1

*Overall diagnoses. ** Total mentioned HPOs. ***Number of individuals with previous hospitalizations

Most confirmed diagnoses were etiological (*n* = 5185; 52.2%), with clinical diagnoses accounting for the remaining cases (*n* = 4743; 47.8%). Among the cases with an etiological diagnosis, most were confirmed through biochemical (*n* = 2164; 42.5%), molecular (*n* = 1574; 30.9%), and cytogenetic (*n* = 691; 13.6%) diagnostic methods (Fig. 1b). The primary funder for the diagnostic tests was the SUS (84.2%).

On average, 2.85 HPOs were reported per case. The most frequent signs and symptoms were global developmental delay (HP:0001263; *n* = 1246), seizure (HP:0001250; *n* = 734), and short stature (HP:0004322; *n* = 678; Table 2). The median age at symptom onset was 0.8 years (IQR: 0–9; mean: 9.2), with a median age of 1 year for confirmed cases and 0.8 years for suspected diagnoses (Table 3). Only 17.8% of participants experienced symptom onset after the age of 18 years (*n* = 1638).

**Table 3:** Comparative analysis based on diagnostic status

	Confirmed diagnosis (N = 7931)	Suspected diagnosis (N = 2450)	Undiagnosed (N = 2177)	Significance
	Median (IQR)	Median (IQR)	Median (IQR)	*P* value
Age (years) (N = 12,159)	18 (9–37)	13 (6–26)	11 (6–18)	< 0.0001*
Age of symptom onset (years) (N = 9328)	1 (0–14)	0.8 (0–8)	0.2 (0–2)	< 0.0001*
Age at first evaluation at the center (years) (N = 11,546)	7.3 (0.7–26.8)	6.5 (1.3–17.4)	3.8 (0.9–10.8)	< 0.0001*
Age at first evaluation in the specialty (years) (N = 11,277)	8.1 (1.1–27.3)	7.6 (1.9–18.5)	5.6 (1.7–12.6)	< 0.0001*
Length of follow-up at the center (years) (N = 11,592)	3.7 (1–9.4)	1.3 (0.2–4.6)	1.8 (0.3–5.5)	< 0.0001*
Length of follow-up in the specialty (years) (N = 11,317)	2.7 (0.6–7.2)	0.6 (0–2.6)	0.5 (0–2.6)	< 0.0001*
Age at confirmatory diagnosis (years) (N=4944)	10.4 (2.1–33.1)	NA	NA	–
Number of previous hospitalizations (N = 4294)	2 (1–4)	1 (1–3)	1 (1–2)	< 0.0001*
Maternal age at birth (years) (N = 4837)	27 (22–33)	27 (22–32)	27 (22–33)	0.332
Paternal age at birth (years) (N = 3996)	31 (25–37)	30 (25.7–37)	31 (25–37)	0.995

	N (%)	N (%)	N (%)	*P* value
*Color or race*				
White	3330 (66.6)	763 (15.3)	907 (18.1)	< 0.0001*
Admixed	3054 (61.2)	1072 (21.5)	863 (17.3)	
Black	425 (69.2)	103 (16.8)	86 (14.0)	
Yellow	45 (64.3)	11 (15.7)	14 (20.0)	
Indigenous	21 (70.0)	5 (16.7)	4 (13.3)	
*Sex*				
Female	4254 (67.4)	1085 (17.2)	971 (15.4)	< 0.0001*
Male	3687 (60.7)	1200 (19.8)	1184 (19.5)	
Undetermined	7 (53.8)	5 (38.5)	1 (7.7)	
*Region of birth*				
Southeast	2516 (66.3)	582 (15.3)	700 (18.4)	< 0.0001*
Northeast	2200 (59.6)	739 (20.0)	753 (20.4)	
South	1258 (72.1)	213 (12.2)	275 (15.7)	
Midwest	828 (61.2)	325 (24.0)	201 (14.8)	
North	321 (47.8)	254 (37.9)	96 (14.3)	
Born in other countries	7 (63.6)	2 (18.2)	2 (18.2)	
*Region of residence*				
Southeast	2688 (66.5)	610 (15.1)	744 (18.4)	< 0.0001*
Northeast	2339 (60.0)	800 (20.5)	758 (19.5)	
South	1658 (76.2)	234 (10.7)	286 (13.1)	
Midwest	906 (61.5)	355 (24.1)	213 (14.4)	
North	299 (46.8)	246 (38.6)	93 (14.6)	
*Family recurrence*				
No	4953 (62.9)	1418 (18.0)	1503 (19.1)	0.030
Yes	1713 (63.5)	531 (19.7)	452 (16.8)	
*Consanguinity*				
No	5487 (61.5)	1697 (19.1)	1734 (19.4)	< 0.0001*
Yes	440 (55.1)	158 (19.8)	200 (25.1)	
*Previous hospitalization*				
No	3789 (62.8)	1143 (19.0)	1099 (18.2)	< 0.0001*
Yes	3583 (70.4)	809 (15.9)	697 (13.7)	
*Death*				
No	7688 (65.0)	2113 (17.9)	2021 (17.1)	0.094
Yes	127 (71.8)	30 (16.9)	20 (11.3)	
*Treatment related to rare disease*				
Yes	5317 (83.9)	620 (9.8)	397 (6.3)	< 0.0001*
No	134 (40.9)	73 (22.3)	121 (36.8)	

Each row corresponds to the total number of valid data, i.e., without considering missing values. In this analysis, each diagnosis was evaluated independently, considering that a participant may have more than one RD diagnosis

P-values marked with * represent statistical significance (P < 0.05)

The diagnosis was made prenatally in only 121 cases (1.2%) and via newborn screening in 979 (9.9%) cases. The median age at confirmatory diagnosis was 10.4 years (IQR: 2.1–33.1) upon excluding prenatal and newborn screening diagnoses (Table 3). The average time from the onset of the first symptom to the diagnostic confirmation was 5.4 ± 7.9 years (*n* = 4583).

### Family history

Family recurrence was reported in 2717 cases (21.6%) and consanguinity in 803 cases (6.4%). Consanguinity rates, expressed as percentages, were significantly higher in the Northeast region (14.0%), followed by the South (7.1%), North (6.5%), Southeast (6%), and Midwest (4.4%; *p* < 0.0001). The mean maternal age at the patient’s birth was 27.7 ± 7.0 years (range: 12–63), and the mean paternal age was 31.7 ± 8.4 years (range: 12–79).

### Treatment

Regarding treatment, 6509 participants (54.3%) received specific therapy to treat their RD or manage its signs and symptoms. The most frequent therapies were drug therapy (*n* = 6108; 55.0%), rehabilitation therapy (*n* = 1739; 15.6%), and dietary therapy (*n* = 976; 8.8%). Drug treatment was initiated at an average age of 22 ± 21.8 years, dietary treatment at 3.2 ± 8.3 years, and rehabilitation at 14.9 ± 19.4 years. The primary funding source for treatments was the SUS (86.7%), which supported 85.6% of the drug treatments, 83.2% of the dietary treatments, and 88.2% of the rehabilitative treatments.

Multi-specialty medical follow-up was reported in 84.0% (*n* = 9864) of participants. Apart from medical genetics, the specialty where most data was collected, neurology was the most consulted specialty, representing 31% of consultations, followed by endocrinology (22.6%), neuropediatrics (21%), and ophthalmology (18.2%).

### Hospitalization and death

A previous hospitalization was recorded for 4922 participants (44.5%). The mean number of hospitalizations was 4.12 ± 14.2 (range: 0–379), with 5% of participants undergoing at least 13 hospitalizations. The most frequent reasons for hospitalization were ICD-10 codes E22.0 (acromegaly and pituitary gigantism; *n* = 189), Q78.0 (osteogenesis imperfecta; *n* = 161), and E84 (CF; *n* = 125; Table 2).

A mortality rate of 1.5% (*n* = 177) was observed in the studied population during the evaluated period. The median age at death was 20.3 years (IQR: 1.6–55.7; mean: 30.3 ± 27.8; range: 0–87.7). The leading causes of death were ICD-10 codes G12.2 (motor neuron disease; *n* = 30), E84 (CF; *n* = 10), and I46 (cardiac arrest; *n* = 7; Table 2). Autopsy was performed in 18 (10.3%) cases.

Table 3 presents comparative data on cases with confirmed diagnoses, suspected diagnoses, and undiagnosed cases based on the investigated characteristics. Details of the statistical results and pairwise comparisons with Bonferroni correction are available in Additional file 4.

## Discussion

This study represents Brazil’s first comprehensive evaluation of RD epidemiology, embodying an innovative approach based on collaborative efforts and a network-based framework. The specialized services network, including NSRSs, contributed to the higher prevalence of PKU and CF diagnoses in this epidemiological survey. Additionally, this study revealed the average duration of the diagnostic odyssey for individuals with RDs in Brazil (5.4 years). Moreover, a substantial portion of patients with RDs were found to remain undiagnosed.

The study population mainly comprised individuals born and residing in Brazil’s Southeast, Northeast, and Southern regions, respectively, which are ranked as the most populous regions in the country [7]. Individuals born in 1750 Brazilian cities were included, representing 31.4% of all national municipalities [7]. Notably, São Paulo city, with 12.4 million inhabitants, has the highest population and contributed the most participants to this study. Higher rates of confirmed diagnoses were found among participants born and residing in the South and Southeast regions of the country compared to other regions, likely due to the greater availability of genetic testing and specialized resources for RDs in these areas, as reported in previous studies [8, 9, 11, 18].

The newborn screening program in Brazil encompasses PKU and CF, contributing to the high frequency of these conditions in this study. The screening also covers congenital hypothyroidism, hemoglobinopathies, congenital adrenal hyperplasia, and biotinidase deficiency [3]. Sickle cell disease was excluded due to its non-rare status in certain states of Brazil, especially among individuals with African ancestry [19]. Medical genetics services’ prevalence may have influenced the lower frequency of congenital hypothyroidism. Upon excluding newborn screening cases, PKU was not the most common diagnosis. A considerable number of cases of CF were not identified through neonatal screening. This may be due to the inclusion of CF in the Brazilian neonatal screening program around 2001 [20] and its complete incorporation may not have occurred immediately. It is also important to consider the possibility of false negatives in the screening process.

Acromegaly emerged as a notable focal point in our study, standing out as one of the three most prevalent conditions in seven participating centers and the most frequent cause of hospitalization in the studied population. This prominence could be attributed to the specialized nature of at least four of these centers, which function as dedicated reference services for acromegaly treatment. This specialization can potentially cause selection bias, as individuals seeking care specifically for acromegaly may contribute disproportionately to the study population from these centers.

In our study, 67 participants had multiple confirmed RD diagnoses, which poses unique challenges and impacts patients physically, emotionally, and financially. With the advancing scope of genomic techniques, having multiple confirmed RD diagnoses is becoming increasingly common [21].

Compared to the 6.4% consanguinity rate observed in our study, previous research indicates variable consanguinity rates in different populations. Leutenegger et al. [22] found inbreeding in various populations around the world, with the highest levels in the Middle East, Central South Asia, and the Americas. A mean consanguinity rate of 0.96% was reported in South America, with higher rates in Venezuela (1.84%) and Brazil (1.60%) [23]. Previous studies have also indicated higher consanguinity rates in the Northeastern region [24]. Factors such as low paternal education and occupation levels were positively associated with consanguinity [23]. The higher consanguinity rates in our study compared to previous studies can be attributed to the population of participants with diagnosed or suspected RDs, including autosomal recessive disorders.

Many participants experienced numerous hospitalizations, especially those with confirmed RD diagnoses, suggesting that these hospitalizations may be related to therapeutic requirements. This observation underscores the complex, multidisciplinary specialized care that individuals with RDs uniquely need and emphasizes the importance of accordingly tailored accessible healthcare. Previous studies have reported the elevated economic burden of hospitalizations for RDs [6] and higher hospitalization rates among patients with metabolic and genitourinary system-related RDs [25]. Additionally, RDs have been previously associated with unfavorable inpatient outcomes, including in-hospital deaths, extended stays, intensive care unit admissions, and 30-day readmissions when compared to an inpatient population without RDs [26].

Some form of instituted therapy was identified more frequently among individuals with confirmed RD diagnoses. Participants with confirmed RD diagnoses may have received more frequent therapy due to selection bias, reflecting possibly more severe symptoms and referrals to specialized centers. Disease severity may have also driven immediate therapy initiation for improved management and outcomes. Ninety-two participants received care from multiple centers, illustrating co-management challenges in complex, multisystem RDs [10, 25]. Our study also emphasized the importance of multidisciplinary care for individuals with RDs. However, it is essential to acknowledge that medical genetics data were not separately collected as a distinct medical specialty. Instead, this specialty was encompassed within the primary care for most cases, where data collection and treatment were conducted.

The SUS plays a vital role in RD diagnosis and treatment. It serves as the primary funder for therapies and diagnostic methods related to RDs. The SUS enables the availability of genetic testing [11], specialized consultations, and treatment options that incorporate the National Committee for Health Technology Incorporation recommendations and enable the subsequent development of clinical guidelines [10, 27]. Working as a network becomes essential to optimize the use of resources and enhance collaboration between institutions.

Five of the 34 participating centers exclusively care for pediatric patients, while the remaining centers offer care to both pediatric and adult patients. This distribution reflects the prevalence of RDs affecting individuals across the age spectrum. Interestingly, our data revealed a median age at symptom onset of 0.8 years, indicating that symptoms typically manifest early in life. Additionally, our findings show that over 80% of individuals experienced symptoms before the age of 18 years, surpassing the figure of 70% reported in a previous study [1]. This difference could be attributed to the participation of dedicated pediatric care centers in our study. Our findings suggest that RD symptoms often present at a younger age, highlighting the need for early diagnosis and intervention, especially in pediatric patients, but continue to pose challenges into adulthood.

The diagnostic odyssey, defined as the time from symptom recognition to a definitive diagnosis [28], averaged 5.4 years, consistent with the figure of 4.8–7.6 years reported in other studies worldwide [29, 30]. Notably, a previous study in Brazil reported that the diagnostic odyssey for mucopolysaccharidosis lasted 4.8 years [31]. Prolonged diagnostic odysseys for RDs often involve disease progression, incorrect diagnoses, invasive procedures, delayed treatment initiation, financial burden, and inappropriate interventions [32].

Despite thousands of described RDs, many remain undiagnosed, subjecting individuals to prolonged, costly diagnostic odysseys across multiple healthcare centers [32]. However, even after such efforts, around 6% and 7% of patients with RDs  in the United States and Australia, respectively, remained undiagnosed even in expert clinical settings [32, 33]. Factors that may explain the higher rates of undiagnosed cases (exceeding 17%) in our study include poor access to molecular diagnostic techniques. A recent study by RARAS reported that molecular diagnostic tests were available in just over half of the participating centers [11]. Most cases with an etiological diagnosis were confirmed through biochemical and molecular methods. Interestingly, while not the primary confirmatory method, cytogenetic testing was the most accessible diagnostic method in the participating centers, according to the same study.

In the comparative analysis, individuals with a confirmed RD diagnosis showed a higher age, longer follow-up duration in specialized centers, and higher number of previous hospitalizations. Specifically, the undiagnosed group may include individuals who are in the diagnostic journey or odyssey and have not yet obtained a confirmed diagnosis. Subsequent investigations within the RARAS initiative will aim to prospectively assess such cases, establishing a national registry of RDs.

The average age at death was 30.3 years, representing a 47-year reduction compared to the Brazilian population’s 2021 life expectancy [34]. In our study, 25% of deaths occurred within the first 1.6 years of life, indicating that RDs significantly impact life expectancy. Previous data suggested that 22% of infant deaths were due to confirmed genetic disorders [35]. Causes of death related to RDs vary and are often documented as complications rather than the underlying disease. Cardiac and respiratory arrests were frequently recorded causes that did not fully represent the primary cause. The accurate documentation of complications and comorbidities is crucial in RDs, offering insights into disease progression and leading to the development of targeted interventions to improve patient care and reduce RD-related mortality [36]. It is important to recognize that undiagnosed cases might also contribute to mortality figures since some individuals may miss the opportunity to receive care in specialized healthcare facilities, leading to an unrealized suspicion of an RD.

While our study provides valuable information, it has limitations, including sample size and potential bias. The estimated population prevalence for RDs ranged from 3.5 to 8.0% [1, 5, 6], suggesting a significantly larger affected population. Considering the Brazilian population, the country’s total number of individuals with RDs would be 550–1200 times larger than the population studied in this project phase [7]. It is essential to note that this study did not include all national healthcare centers, potentially missing patients not evaluated during the study or not receiving care at participating centers. Moreover, the predominance of genetic RDs may have resulted from the specialized expertise and diagnostic resources in genetic centers, leading to selection bias.

This study faced operational limitations related to data sources, including finding, accessing, sharing, and reusing information. A “data quality culture” was promoted to address these issues, emphasizing the need for reliable and comprehensive data. Collectors had diverse backgrounds and digital literacy levels, which could have introduced errors and affected data reliability. Tools, training, support materials, and dedicated channels were provided to mitigate their effects. The complex RD domain made case identification and classification challenging, potentially leading to underreporting and underdiagnosis. Awareness efforts, feedback sessions, outlier identification, case discussions, and standardized data collection protocols were implemented to address this issue [2, 37].

This study revealed appreciable missing data in medical records, which can introduce record-keeping, memory, and registration biases. Missing data in medical records can limit retrospective research, potentially due to registration bias. However, data collection directly from participants in the prospective project phase aims to fill these gaps. A potential contribution of our study is the enhancement of registration methods. By identifying and addressing limitations in data collection and diagnostic terminology classification, we lay the groundwork for more accurate and comprehensive RD registration. This enhancement improves our understanding of RD epidemiology and supports the development of effective public health policies and resource allocation strategies. Standardized data collection protocols and advanced information systems will ensure that future studies and registries capture vital data points, facilitating ongoing RD monitoring and research [2].

Diagnosis data in our study came from three different ontologies, each with limitations regarding disease terminology. While this study's protocol allowed centers to select RD terminology, including ICD-10, it had limitations in RD classification [38, 39]. Accurate RD classification is crucial for efficient healthcare resource allocation and improved analysis for differential diagnosis and clinical decision support. While data were aggregated from the Orphadata database designed for RDs, this database does not encompass all described RDs. In Brazil, ICD-10 remains the classification used by the SUS for diagnosis, hospitalization, and death registration [10, 39]. In the context of HPO terminology, it is noteworthy that the number of HPO terms may have been underestimated due to the limitation of five terms per case.

Future research within the RARAS will encompass the diagnostic and treatment journey of participants with multiple confirmed diagnoses, explore specific therapies and the duration of hospitalizations, investigate the correlation between diagnostic ontologies, and examine population genetics. Other research avenues include exploring the relationship between parental age and RDs and examining the correlations of diagnoses with available diagnostic methods at each center.

We also identified challenges in finding a minimal data set (MDS) that applied to Brazilian patients with RDs. To address this issue, we conducted a systematic review to create a comprehensive MDS for future project phases [40, 41]. Standardizing data collection through an MDS is critical for accurately identifying RDs and optimizing diagnostic and treatment processes, particularly in resource-limited settings. Validating it as a national tool for epidemiological tracking and analysis is essential for structuring health information systems and guiding more effective public health policies. Further research phases are required to refine prevalence estimates and comprehensively understand specific RDs and their impact on the Brazilian population by including a broader range of healthcare facilities. This retrospective analysis did not address factors such as participants’ socioeconomic status, referral sources, or willingness to participate in other studies. However, these variables became part of the data collection protocol and will be examined in forthcoming studies.

The perspectives presented here shed light on the future research directions derived from our study, fostering further advancements in the field. These data can support future studies and ultimately lead to improvements in RD diagnosis, treatment, and management. Understanding the magnitude of RDs is crucial for effective resource allocation, policy development, and the provision of appropriate healthcare services for affected individuals [3, 5].

This multicenter study presents the initial nationwide data on the care provided to individuals with RDs in Brazil, highlighting the importance of collaboration between specialized services. Reliable epidemiological data will support public health approaches, including population impact assessment, cost evaluation, and improved RD management, and facilitate clinical trial development [5]. This study also emphasizes the vital role of the collected information in shaping public policies while identifying limitations such as data gaps and constrained terminologies for disease classification. Until this study was performed, our understanding of RDs in Brazil, except for specific disorders, was limited by a lack of comprehensive evidence. Establishing a national network, including data collection infrastructure, marked a significant step towards advancing the understanding of RDs in Brazil and addressing this gap.

The longitudinal and prospective continuation of this study is necessary and currently underway, with the expectation that it will impact health policy for RDs regarding resource allocation and improving the quality of life of affected individuals. The results of our study also provide valuable guidance for the refinement of data collection forms and instruments, thereby enhancing the effectiveness and accuracy of information related to RDs in Brazil.

## Supplementary Information

Additional file 1. Map of participating centers

Additional file 2. The ten most frequent RD diagnoses in RARAS and the applied coding

Additional file 3. Top three diagnostic codes and their corresponding counts and percentages at each participating center

Additional file 4. Post-test analysis of demographic factors and medical outcomes across distinct diagnostic statuses

## Data Availability

Data analyzed in this study are available interactively through the Brazilian Online Atlas of RD (RARASBR; 10.25504/FAIRsharing.d7b6c8) [42] and LattesData [13]. For any further inquiries, please contact the corresponding author.
