# Supplementary figures and images for: Epidemiological characterization of rare diseases in Brazil: A retrospective study of the Brazilian Rare Diseases Network
Source: Orphanet J Rare Dis. 2024 Oct 30;19:405. doi: 10.1186/s13023-024-03392-7 (PMC11523578; doi:10.1186/s13023-024-03392-7)

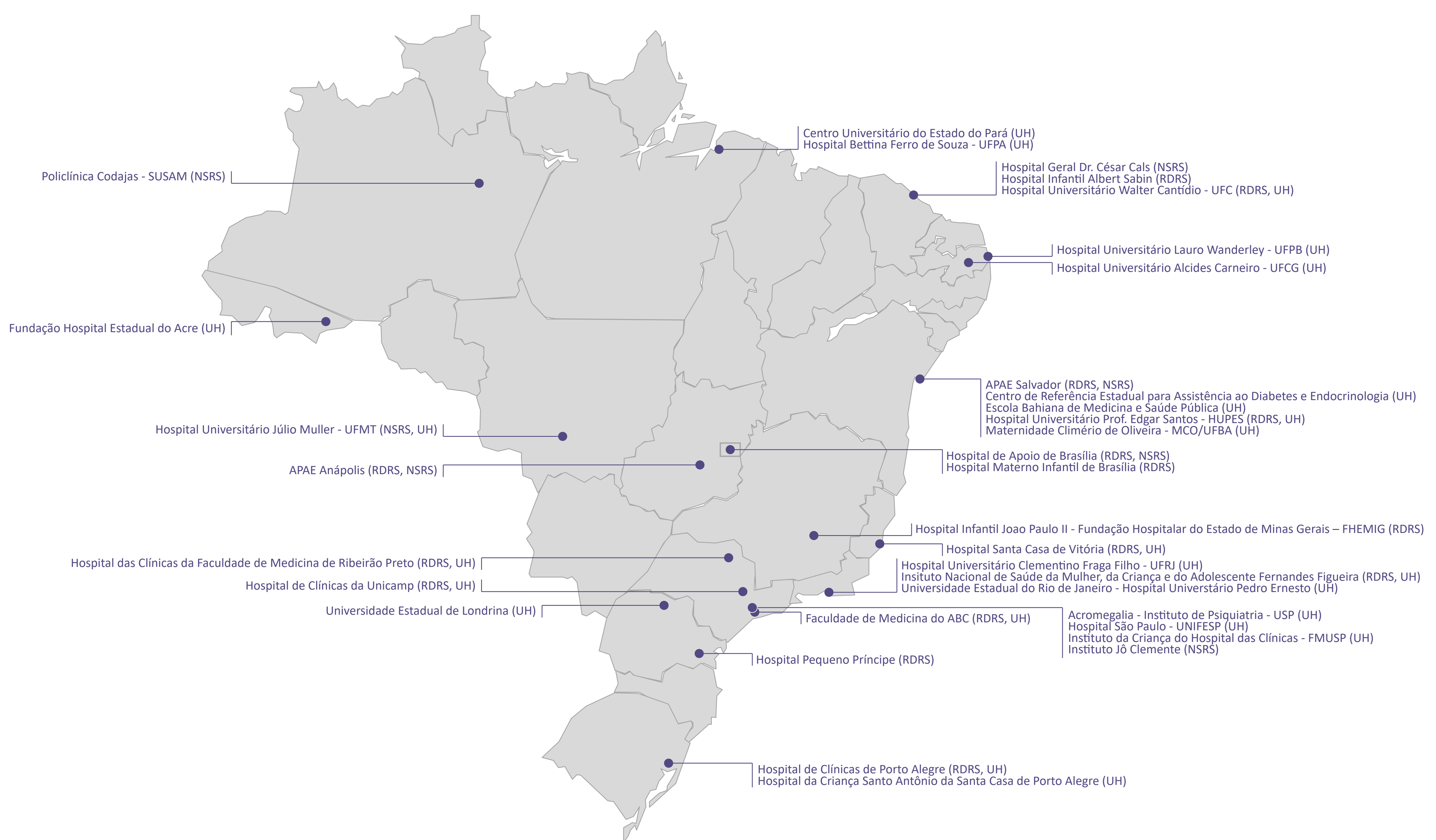

Supplement: Supplementary file 1 — Additional file 1. Map of participating centers [file 13023_2024_3392_MOESM1_ESM.pdf]
