# Supplementary material for: Epidemiological characterization of rare diseases in Brazil: A retrospective study of the Brazilian Rare Diseases Network
Source: Orphanet J Rare Dis. 2024 Oct 30;19:405. doi: 10.1186/s13023-024-03392-7 (PMC11523578; doi:10.1186/s13023-024-03392-7)
Supplement: Supplementary file 2 — Additional file 2. The ten most frequent RD diagnoses in RARAS and the applied coding [file 13023_2024_3392_MOESM2_ESM.pdf]

Additional file 2 - Table: The ten most frequent RD diagnoses in RARAS and the applied coding.

Disorder	Coding and description	N
**Phenylketonuria (n=623)**	ORPHA - 716 - Phenylketonuria	533
	ICD-10 - E70.0 - Classical phenylketonuria	57
	OMIM - 261600 - Phenylketonuria	30
	ICD-10 - E70.1 - Other hyperphenylalaninemias	3
**Cystic Fibrosis (n=506)**	ICD-10 - E84.9 - Cystic fibrosis, unspecified	192
	ORPHA - 586 - Cystic fibrosis	124
	ICD-10 - E84.0 - Cystic fibrosis with pulmonary manifestations	89
	ICD-10 - E84.8 - Cystic fibrosis with other manifestations	66
	OMIM - 219700 - Cystic fibrosis	23
	ICD-10 - E84.1 - Cystic fibrosis with intestinal manifestations	12
**Acromegaly (n=382)**	ICD-10 - E22.0 - Acromegaly and pituitary gigantism	278
	ORPHA - 963 - Acromegaly	101
	OMIM - 102200 - 300943 - Acromegaly due to Pituitary Adenoma	3
**Osteogenesis Imperfecta (n=360)**	ICD-10 - Q78.0 - Osteogenesis imperfecta	179
	ORPHA - 666 - Osteogenesis imperfecta	59
	ORPHA - 216796 - Osteogenesis imperfecta type 1	56
	ORPHA - 216820 - Osteogenesis imperfecta type 4	33
	ORPHA - 216812 - Osteogenesis imperfecta type 3	24
	OMIM - 610682 - 610915 - 610967 - 610968 - 613848 - 613849 - 613982 - 614856 - 615066 - 615220 - 616229 - 616507 - 166200 - 166210 - 166220 - 166230 - 259420 - 259440 - Osteogenesis imperfecta	4
	ORPHA - 216804 - Osteogenesis imperfecta type 2	2
	ORPHA - 2771 - Bruck syndrome	1
	ORPHA - 216828 - Osteogenesis imperfecta type 5	1
	OMIM - 610967 - Osteogenesis imperfecta type 5	1
**Dystrophinopathy (n=278)**	ORPHA - 98896 - Duchenne muscular dystrophy	220
	ORPHA - 98895 - Becker muscular dystrophy	37
	ORPHA - 206546 - Symptomatic form of muscular dystrophy of Duchenne and Becker in female carriers	11
	OMIM - 310200 - Muscular dystrophy, Duchenne type	10
**Congenital adrenal hyperplasia (n=275)**	ICD-10 - E25.0 - Congenital adrenogenital disorders associated with enzyme deficiency	122
	ORPHA - 315306 - Classic congenital adrenal hyperplasia due to 21-hydroxylase deficiency, salt wasting form	113
	ORPHA - 315311 - Classic congenital adrenal hyperplasia due to 21-hydroxylase deficiency, simple virilizing form	29
	ORPHA - 418 - Congenital adrenal hyperplasia	6
	ORPHA - 90793 - Congenital adrenal hyperplasia due to 17-alpha-hydroxylase deficiency	2

	ORPHA - 90794 - Classic congenital adrenal hyperplasia due to 21-hydroxylase deficiency	1
	OMIM - 201910 - Adrenal hyperplasia, congenital, due to 21-hydroxylase deficiency	1
	OMIM - 201710 - 201810 - 201910 - 202010 - 202110 - 613571 - Congenital adrenal hyperplasia	1
**Neurofibromatosis (n=271)**	ORPHA - 636 - Neurofibromatosis type 1	174
	ICD-10 - Q85.0 - Neurofibromatosis (nonmalignant)	70
	OMIM - 162200 - 162210 - 613675 - Neurofibromatosis type 1	18
	ORPHA - 637 - Full NF2 related schwannomatosis	6
	ORPHA - 363700 - Neurofibromatosis type 1 due to NF1 mutation or intragenic deletion	3
**Mucopolysaccharidosis (n=225)**	ICD-10 - E76.1 - Mucopolysaccharidosis, type II	34
	ORPHA - 583 - Mucopolysaccharidosis type 6	31
	ORPHA - 580 - Mucopolysaccharidosis type 2	31
	ORPHA - 579 - Mucopolysaccharidosis type 1	20
	ORPHA - 309297 - Mucopolysaccharidosis type 4A	20
	OMIM - 253000 - Mucopolysaccharidosis type 4A	15
	ORPHA - 582 - Mucopolysaccharidosis type 4	14
	OMIM - 253200 - Mucopolysaccharidosis type 6	13
	ICD-10 - E76.0 - Mucopolysaccharidosis, type 1	12
	ORPHA - 581 - Mucopolysaccharidosis type 3	12
	ORPHA - 584 - Mucopolysaccharidosis type 7	6
	ORPHA - 79270 - Sanfilippo syndrome type B	6
	OMIM - 607014 - 607015 - 607016 - Mucopolysaccharidosis type I	3
	OMIM - 309900 - Mucopolysaccharidosis type II	3
	ICD-10 - E76.3 - Mucopolysaccharidosis, unspecified	2
	ICD-10 - E76.2 - Other mucopolysaccharidoses	1
	ORPHA - 79269 - Sanfilippo syndrome type A	1
	OMIM - 252900 - 252920 - 252930 - 252940 - Mucopolysaccharidosis 3	1
**Amyotrophic lateral sclerosis (n=211)**	ORPHA - 803 - Amyotrophic lateral sclerosis	208
	ORPHA - 300605 - Juvenile amyotrophic lateral sclerosis	2
	OMIM - 611637 - Primary lateral sclerosis, Adult 1	1
**Turner Syndrome (n=197)**	ORPHA - 881 - Turner syndrome	176
	ICD-10 - Q96 - Turner's syndrome	20
	ICD-10 - Q96.9 - Turner's syndrome, unspecified	1
