# Supplementary material for: Epidemiological characterization of rare diseases in Brazil: A retrospective study of the Brazilian Rare Diseases Network
Source: Orphanet J Rare Dis. 2024 Oct 30;19:405. doi: 10.1186/s13023-024-03392-7 (PMC11523578; doi:10.1186/s13023-024-03392-7)
Supplement: Supplementary file 3 — Additional file 3. Top three diagnostic codes and their corresponding counts and percentages at each participating center [file 13023_2024_3392_MOESM3_ESM.pdf]

Additional file 3 - Table: Top Three Diagnostic Codes and Their Corresponding Counts and Percentages at Each Participating Center

Participating Center - City/State	Disease Code	Condition	N	%*
**Southeast region**				
**Acromegalia - Instituto de Psiquiatria - USP - São Paulo/SP**	ICD-10:E22.0	Acromegaly and pituitary gigantism	115	95.0
	ICD-10:E23.0	Hypopituitarism	5	4.1
	ICD-10:E23.2	Central diabetes insipidus	1	0.8
**Faculdade de Medicina do ABC - Santo André/SP**	ORPHA:1572	Common variable immunodeficiency	7	12.0
	ORPHA:586	Cystic fibrosis	4	6.9
	ICD-10:G93.4	Other and unspecified encephalopathy	3	5.2
**Hospital das Clínicas da Faculdade de Medicina de Ribeirão Preto - Ribeirão Preto/SP**	ORPHA:803	Amyotrophic lateral sclerosis	102	9.2
	ORPHA:963	Acromegaly	96	8.7
	ORPHA:98896	Duchenne muscular dystrophy	82	7.4
**Hospital Infantil João Paulo II - Belo Horizonte/MG**	ICD-10:E84.9	Cystic fibrosis, unspecified	88	52.4
	ICD-10:E84.0	Cystic fibrosis with pulmonary manifestations	45	26.8
	ICD-10:E84.8	Cystic fibrosis with other manifestations	33	19.6
**Hospital Santa Casa de Vitória - Vitória/ES**	ICD-10:D84.1	Defects in the complement system	66	100.0
**Hospital São Paulo - São Paulo/SP**	ORPHA:324	Fabry disease	27	27.0
	ORPHA:77259	Gaucher disease type 1	13	13.0
	ORPHA:579	Mucopolysaccharidosis type 1	9	9.0
**Hospital Universitário Clementino Fraga Filho / UFRJ - Rio de Janeiro/RJ**	ICD-10:D84.1	Defects in the complement system	71	98.6
	ORPHA:91378	Hereditary angioedema	1	1.4
**Hospital Universitário Pedro Ernesto - Rio de Janeiro/RJ**	ICD-10:E22.0	Acromegaly and pituitary gigantism	28	13.0
	ICD-10:Q99.2	Fragile X chromosome	8	3.7
	ICD-10:Q85.1	Tuberous sclerosis	6	2.8
**Instituto da Criança / USP - São Paulo/SP**	ORPHA:567	22q11.2 deletion syndrome	9	8.9
	OMIM:162200 - 162210 - 613675	Neurofibromatosis type 1	4	3.9
	OMIM:194050	Williams syndrome	4	3.9
**Instituto Jô Clemente - São Paulo/SP**	ORPHA:716	Phenylketonuria	389	100.0
**Instituto Nacional de Saúde da Mulher, da Criança e do Adolescente Fernandes Figueira IFF/Fiocruz - Rio de Janeiro/RJ**	ICD-10:E84.8	Cystic fibrosis with other manifestations	30	10.1
	ICD-10:E84.0	Cystic fibrosis with pulmonary manifestations	25	8.4
	ICD-10:Q78.0	Osteogenesis imperfecta	21	7.0
**Universidade Estadual de Campinas, UNICAMP - Campinas/SP**	ORPHA:636	Neurofibromatosis type 1	41	7.1
	ORPHA:908	Fragile X syndrome	24	4.1
	ORPHA:251510	46,XY partial gonadal dysgenesis	20	3.4
**Northeast region**				

**APAE - Salvador/BA**	ICD-10:E25.0	Congenital adrenogenital disorders associated with enzyme deficiency	58	35.2
	ICD-10:E70.0	Classical phenylketonuria	36	21.8
	ORPHA:315306	Classic congenital adrenal hyperplasia due to 21-hydroxylase deficiency, salt wasting form	19	11.5
**CEDEBA - Salvador/BA**	ICD-10:E22.0	Acromegaly and pituitary gigantism	77	100.0
**Escola Bahiana de Medicina e Saúde Pública - Salvador/BA**	ORPHA:289326	Tropical spastic paraparesis	70	15.1
	ORPHA:803	Amyotrophic lateral sclerosis	64	13.8
	ICD-10:G71.0	Muscular dystrophy	46	9.9
**Hospital Geral Dr. César Cals - Fortaleza/CE**	ICD-10:K22.0	Achalasia of cardia	27	9.3
	ICD-10:O01.0 - O01.1 - O01.9	Hydatidiform mole	14	4.8
	ORPHA:589	Myasthenia gravis	12	4.1
**Hospital Infantil Albert Sabin - Fortaleza/CE**	OMIM:261600	Phenylketonuria	30	8.3
	ICD-10:Q78.0	Osteogenesis imperfecta	24	6.6
	ORPHA:716	Phenylketonuria	17	4.7
**Hospital Universitário Alcides Carneiro/UFCG/EBSERH - Campina Grande/PB**	ORPHA:881	Turner syndrome	12	8.0
	ICD-10:E22.0	Acromegaly and pituitary gigantism	10	6.7
	OMIM:253000	Mucopolysaccharidosis type 4	10	6.7
**Hospital Universitário Lauro Wanderley/UFPB/EBSERH - João Pessoa/PB**	ICD-10:Q87.4	Marfan syndrome	8	9.0
	ICD-10:Q87.1	Congenital malformation syndromes predominantly associated with short stature	7	7.8
	ICD-10:Q85.0	Neurofibromatosis (nonmalignant)	5	5.6
**Hospital Universitário Prof. Edgar Santos / UFBA - Salvador/BA**	ICD-10:Q78.0	Osteogenesis imperfecta	89	6.2
	ORPHA:881	Turner syndrome	59	4.1
	ICD-10:E25.0	Congenital adrenogenital disorders associated with enzyme deficiency	48	3.3
**Hospital Universitário Walter Cantídio / UFC / EBSERH - Fortaleza/CE**	ORPHA:881	Turner syndrome	15	11
	ORPHA:98896	Duchenne muscular dystrophy	6	4.4
	ORPHA:648	Noonan syndrome	4	2.9
**Maternidade Climério de Oliveira - Salvador/BA**	ICD-10:Q92.9	Trisomy and partial trisomy of autosomes, unspecified	1	50.0
	OMIM:265380	Congenital alveolar capillary dysplasia	1	50.0
**South region**				
**Hospital da Criança Santo Antônio / Irmandade da Santa Casa de Misericórdia de Porto Alegre - Porto Alegre/RS**	ORPHA:567	22q11.2 deletion syndrome	25	19.8
	ORPHA:636	Neurofibromatosis type 1	8	6.3
	ORPHA:199	Cornelia de Lange syndrome	5	3.9
**Hospital de Clínicas de Porto Alegre - Porto Alegre/RS**	ORPHA:636	Neurofibromatosis type 1	59	6.9
	ORPHA:716	Phenylketonuria	40	4.6
	ORPHA:98757	Spinocerebellar ataxia type 3	35	4.1
**Hospital Pequeno Príncipe - Curitiba/PR**	ICD-10:Q99.8	Other specified chromosome abnormalities	29	7.9

	ORPHA:98896	Duchenne muscular dystrophy	28	7.6
	OMIM:219700	Cystic fibrosis	22	6.0
**Universidade Estadual de Londrina - Londrina/PR**	ORPHA:315306	Classic congenital adrenal hyperplasia due to 21-hydroxylase deficiency, salt wasting form	53	9.9
	ICD-10:E22.0	Acromegaly and pituitary gigantism	34	6.3
	ORPHA:803	Amyotrophic lateral sclerosis	22	4.1
**Midwest region**				
**APAE - Anápolis/GO**	ICD-10:Q87.1	Congenital malformation syndromes predominantly associated with short stature	16	11.7
	ICD-10:F79.9	Unspecified mental retardation Without mention of impairment of behaviour	9	6.5
	ICD-10:F80.9	Developmental disorder of speech and language, unspecified	9	6.5
**Hospital de Apoio de Brasília - Brasília/DF**	OMIM:301500	Fabry disease	13	6.6
	OMIM:162200 - 162210 - 613675	Neurofibromatosis type 1	9	4.6
	OMIM:230800	Gaucher disease	9	4.6
**Hospital Materno Infantil - Brasília/DF**	ORPHA:648	Noonan syndrome	6	3.1
	ORPHA:881	Turner syndrome	5	2.5
	OMIM:130650	Beckwith-Wiedemann syndrome	4	2.0
**Hospital Universitário Júlio Muller/UFMT/EBSERH - Cuiabá/MT**	ORPHA:442	Congenital hypothyroidism	151	21.0
	ORPHA:232	Sickle cell anemia	35	4.9
	ORPHA:586	Cystic fibrosis	20	2.8
**North region**				
**CESUPA - Belém/PA**	ICD-10:E22.0	Acromegaly and pituitary gigantism	10	71.4
	ICD-10:E24.0 - D35.2	ACTH-dependent Cushing syndrome	3	21.4
	ICD-10:E25.0	Congenital adrenogenital disorders associated with enzyme deficiency	1	7.1
**Policlínica Codajás - Manaus/AM**	ORPHA:716	Phenylketonuria	22	34.9
	ORPHA:315306	Classic congenital adrenal hyperplasia due to 21-hydroxylase deficiency, salt wasting form	13	20.6
	ORPHA:586	Cystic fibrosis	8	12.7
**Fundação Hospital Estadual do Acre - Rio Branco/AC**	ICD-10:Q85.0	Neurofibromatosis (nonmalignant)	5	13.9
	OMIM:540000	Mitochondrial myopathy, encephalopathy, lactic acidosis, and stroke-like episodes, MELAS Syndrome	3	8.3
	ICD-10:Q05.2	Lumbar spina bifida with hydrocephalus	2	5.5
**Hospital Bettina Ferro de Souza- Instituto de Ciências da Saúde/UFPA/EBSERH - Belém/PA**	ORPHA:98896	Duchenne muscular dystrophy	31	7.4
	ICD-10:Q99.8	Other specified chromosome abnormalities	13	3.1
	ORPHA:904	Williams syndrome	12	2.9

*Relative to the total participants within the center.
